# Electrochemical Stripping Analysis at Paper-Based (Bio)Sensors: Current State-of-the-Art and Prospects

**DOI:** 10.3390/s26092819

**Published:** 2026-04-30

**Authors:** Christos Kokkinos, Anastasios Economou

**Affiliations:** Department of Chemistry, National and Kapodistrian University of Athens, 157 71 Athens, Greece; christok@chem.uoa.gr

**Keywords:** paper-based devices, electrochemical stripping analysis, (bio)sensors

## Abstract

Paper-based devices (PADs) have gained increasing attention over the last few years as portable, low-cost and disposable (bio)sensors for point-of-care and on-site analysis. Electrochemistry is a particularly attractive detection mode in PAD assays thanks to its sensitivity and compatibility with portable instrumentation. In particular, electrochemical stripping analysis (ESA) is one of the most sensitive electroanalytical techniques, and, therefore, is suitable for trace assays required in environmental monitoring, clinical diagnostics and food control. Coupling paper as a functional platform with the exceptional sensitivity of ESA creates a powerful analytical tool for trace metals and (bio)sensing. This perspective briefly outlines the current state-of-the art in the field of paper-based (bio)sensors using ESA. It describes the principle of ESA, illustrates different strategies for on-paper electrode fabrication and modification and demonstrates representative applications to trace metal analysis and biosensing. Finally, limitations are identified and future prospects are discussed.

## 1. Introduction

Paper-based (bio)sensors have attracted increasing interest as innovative analytical platforms thanks to their potential for decentralized (bio)chemical analysis [1,2,3]. Paper-based devices (PADs) exploit paper as a functional platform in order to perform (bio)analysis relying on the advantageous mechanical properties of cellulose paper (low thickness, flexibility, fibrous matrix, and lightness), and its wide availability [3]. Unlike conventional analytical systems that often require sophisticated benchtop instrumentation, trained personnel and well-equipped laboratories, PADs are fabricated in a cost-effective manner from inexpensive materials using simple manufacturing techniques [4,5]. The porous paper cellulose network can be exploited to store reagents, promotes passive fluid transport without the need for external pumps and provides a versatile matrix for immobilizing reagents, nanomaterials, and biorecognition elements [3,6,7,8,9,10]. These features make paper-based sensors particularly attractive for decentralized testing, field analysis, and point-of-care diagnostics in various fields such as in environmental monitoring [11], clinical analysis [12], food safety [13] and forensics [14].

Colorimetry is the most widely used detection format in PADs since it can be used to detect a wide variety of targets enabling simple and instrument-free analysis via either visual readout or the use of smartphones [1,15]. On the other hand, electrochemical detection provides higher sensitivity, and increased scope for quantitation and device miniaturization and integration [16,17,18,19]. Recent advances in miniaturized electrochemical instrumentation with low power requirements have accelerated the adoption of electrochemical detection in paper-based analytics [1].

Within this context, electrochemical stripping analysis (ESA) stands out as the most sensitive electroanalytical technique [20,21,22]. This feature makes ESA extremely useful in trace analysis, which is required in fields such as environmental monitoring (e.g., heavy metals detection [20,22]) and clinical diagnostics (protein biomarkers and DNA assays [23]). Additionally, the high sensitivity of ESA is particularly advantageous for paper-based sensors, which often operate with microliter-scale sample volumes and require further signal amplification. The synergy between paper-based platforms and electrochemical stripping analysis has enabled the detection of ultra-trace levels of various analytes [24,25].

A requirement for implementing electrochemical detection in PADs is the use of a suitable set of electrodes. Paper substrates can be engineered to incorporate electrochemical transducers using a wide host of methodologies [16,17,18]. These transducers are often properly modified to enhance analyte accumulation and stripping efficiency, to suppress interferences, and to improve conductivity and target affinity [8,26,27,28]. These modifications are particularly important for stripping-based measurements, as electrode surface properties strongly influence the preconcentration and stripping processes.

This perspective describes the operational principle of ESA, illustrates different strategies for on-paper electrode fabrication and modification, discusses typical PAD configurations and demonstrates representative applications to trace metal analysis and biosensing. Finally, the limitations are identified and the future prospects are discussed.

## 2. Electrochemical Stripping Analysis

ESA is a highly sensitive and widely used electroanalytical technique for the determination of trace and ultra-trace levels of chemical species in a variety of matrices. ESA is a two-step process: the first stage involves accumulation/preconcentration of the target(s) on a suitable working transducer while, in the second step, the target(s) is electrochemically removed from the working electrode by reduction or oxidation producing a signal whose magnitude is proportional to the target(s) concentration in the sample [21].

Owing to its inherent preconcentration capability, ESA offers detection limits that are often several orders of magnitude lower than those achievable with conventional electrochemical methods. Since its introduction, ESA has become a key tool in environmental monitoring, clinical analysis, food safety, and industrial quality control [20,22,23,29].

**Figure 1:**
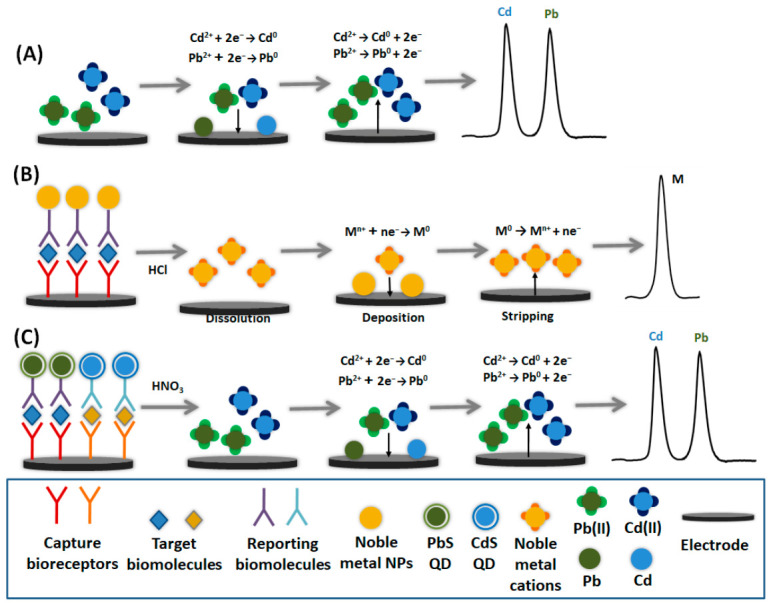
Principle of ESA for the determination of: (**A**) metal cations by ASV after electrolytic accumulation, (**B**) biomolecules using noble metal nanoparticles (Ag, Au) as labels and ASV, (**C**) biomolecules using quantum dots (QDs) as labels and ASV.

Depending on the mechanism of the accumulation and stripping steps, different variants of ESA have been developed. Typically, the target analyte(s) are preconcentrated onto the working electrode by electrochemical reduction, oxidation, or adsorption [21]. Electrolytic accumulation is the most widely used method, commonly employed for the detection of metal ions [20] (Figure 1A). On the other hand, adsorptive accumulation relies on the adsorption of the analyte or surface-active analyte–ligand complexes onto the electrode surface prior to stripping [21]. Regarding the stripping step, it is commonly performed by applying a voltammetric scan at the working electrode; depending on the direction of the potential scan, anodic stripping voltammetry (ASV) and cathodic stripping voltammetry (CSV) can be distinguished [22]. Another approach is to oxidize or reduce the accumulated species using a chemical agent or a constant current (potentiometric stripping analysis (PSA)) [30].

The versatility of these approaches allows ESA to be adapted for a wide range of inorganic and organic analytes. A key advantage of electrochemical stripping analysis lies in its excellent sensitivity and selectivity, which can be further enhanced through careful choice of electrode materials, supporting electrolytes, and complexing agents [20,21]. Moreover, the technique requires relatively simple instrumentation, making it attractive for both laboratory-based and field applications. Advances in electrode design—including the use of nanostructured materials, modified electrodes, and environmentally friendly alternatives to mercury-based electrodes—have significantly improved the performance and applicability of ESA [29].

## 3. Fabrication and Modification of PADs for ESA

The fabrication of electrochemical PADs (ePADs) has become an important area of research due to the growing demand for low-cost, portable, and user-friendly sensing platforms. ePADs combine the inherent advantages of paper substrates—such as capillary-driven fluid transport, disposability, light weight, and environmental friendliness—with the high sensitivity and quantitative capabilities of electrochemical detection. These characteristics make ePADs particularly attractive for point-of-care diagnostics, environmental monitoring, food safety, and on-site analysis in resource-limited settings [18,31,32].

Different types of filter, chromatographic or office paper have been used as PAD substrates [33,34,35]. Paper can be easily patterned to define microfluidic channels and reaction zones using simple and scalable techniques such as: wax printing [36,37,38,39,40,41,42], inkjet printing with hydrophobic inks [43], laser cutting, pen-drawing with hydrophobic ink [44,45,46], lacquer-spraying [47], screen printing, and photolithography [4,48]. These fabrication methods allow precise control over fluid flow and sample distribution while maintaining low production costs and compatibility with mass manufacturing. More elaborate designs allow the fabrication of origami configurations [37] or multi-layered devices [39,49] that enable sampling, solution manipulation and detection within a fully integrated device. Furthermore, with proper design, reservoirs for storing reagents can be created within the porous structure of paper alleviating the need for external addition of solutions [50,51,52]. In biosensing applications, further modification of the paper surface with bioreceptors (i.e., enzymes, antibodies, aptamers, or nucleic acids) is necessary to promote signal-generating interaction with the target molecules. These biorecognition moieties can be immobilized directly onto the paper or electrode surface using physical adsorption, covalent bonding, or entrapment within polymeric matrices [32,53].

A critical component in the fabrication of electrochemical PADs is the integration of electrodes onto the paper substrate. Typically for ESA, a three-electrode configuration—comprising working electrode, reference electrode, and counter electrode—is employed. Carbon-based materials are widely used due to their low cost, wide availability, chemical stability, and good electrochemical performance. Carbonaceous electrodes can be fabricated using various approaches, including screen printing with carbon conductive inks [31,39,44,48,54,55,56,57], inkjet printing of conductive inks [58], pen-plotting with conductive materials [45], stencil printing [59,60] or even hand-drawing with graphite pencils [16,17,18,61]. An interesting approach for the fabrication of carbonaceous electrodes on-paper is the in situ laser treatment of cellulose leading to the formation of conductive patterns via carbonization [62]. Other carbon materials used in conjunction with ePADs include carbon paper [49,63,64,65], graphite foil [66] and boron doped diamond (BDD) [37,67]. Sputtering of metal films (gold, bismuth or tin) on paper results in a thin and uniform electrode surface with exceptional sensitivity towards heavy metals [42,68,69]. Finally, indium-tin oxide (ITO) has been reported as an electrode material in ESA [63,70].

To improve analytical performance, electrode surfaces in electrochemical PADs are frequently modified with functional materials. These modifications aim to enhance electron transfer, to increase the effective surface area, and to promote selective interaction with the target analytes [8,26,27,28]. The modification of electrodes plays a critical role in enhancing the performance of ePADs, particularly for applications involving ESA. Since stripping techniques rely on efficient analyte preconcentration and well-defined redox processes at the electrode surface, the physicochemical properties of the working electrode—such as surface area, conductivity, chemical affinity, and catalytic activity—directly influence sensitivity, selectivity, and detection limits. The incorporation of electrode modifiers also enhances analyte preconcentration, a key step in stripping analysis. Functional coatings can promote selective adsorption or complexation of target species, leading to improved accumulation during the deposition step. In adsorptive stripping analysis, the use of ligand-modified surfaces is especially important, as analyte–ligand complexes are immobilized on the electrode prior to the stripping step, enabling the detection of both inorganic and organic analytes at trace levels. Although bare carbon or metal electrodes have been used occasionally in ESA [39,66], it is generally accepted that they usually provide insufficient performance for ultra-trace analysis. Mercury is an excellent electrode modifier [51,71,72] for trace metal determination but its toxicity prevents its widespread use. Instead, bismuth films [41,44,48,50,54,55,57,60,64] and other mercury-free metal films (e.g., Cu [47,73]), as well as “green” metal nanoparticles (e.g., gold [37,38,56,74]), are being increasingly used as electrode coatings because they form alloys with target metal ions enabling sensitive detection while maintaining environmental and user safety. On the other hand, solid “green” precursors (e.g., bismuth citrate [46], Bi_2_O_3_ [60] or Sb_2_O_3_) [75]) are particularly attractive for the bulk modification of electrodes since they can be reduced in situ to metal films during the analysis. Carbonaceous nanomaterials such as graphene [76] and carbon nanotubes (CNTs) [40,77] are employed to decorate electrodes, essentially to improve electrode conductivity. In addition, a variety of hybrid nanostructured materials have been used to modify electrodes including: reduced graphene oxide (rGO)-carbon black (CB) [43]; GO–multi-wall CNTs (MWCNTs) [78]; carbon nanofibers (CNFs) or rGO with gold nanoparticles (AuNPs) [79]; metal-organic framework (MOF)- AuNPs [49]; rGO-AuNPs [80]; selenium nanoparticles (SeNPs)-AuNPs [65]; rGO-CeO_2_ [81]; MB/Ti_3_C_2_T_x_/MWCNTs [82]; and graphene-bismuth nanoparticles (BiNPs) [83]. Also, polymer films (e.g., Nafion [50] and graphene-polyaniline (PANI) [36]), as well as chelating agents (such as CB-dimethylglyoxime (DMG) [58] and 10-phenanthroline/Nafion [76]) have been employed to selectively bind metal ions or electroactive complexes on paper-based electrodes.

Most PADs and ePADs are single-use sensors since it is usually difficult, time-consuming or impractical to regenerate their surfaces. However, this drawback is offset by the potential to mass-produce them rapidly at low cost (typical fabrication cost is 0.07–1 USD per device) [84,85].

## 4. Determination of Trace Metals and Organics at PADs by ESA

Trace metal analysis on ePADs represents a convergence of electroanalytical chemistry and low-cost microfluidic platforms aimed at decentralized and in situ metal monitoring [24,86]. The compatibility of ESA with paper substrates has enabled detection limits in the low parts-per-billion or lower range for metals such as Pb(II), Cd(II), Hg(II) and metalloids such as As(III).

**Figure 2:**
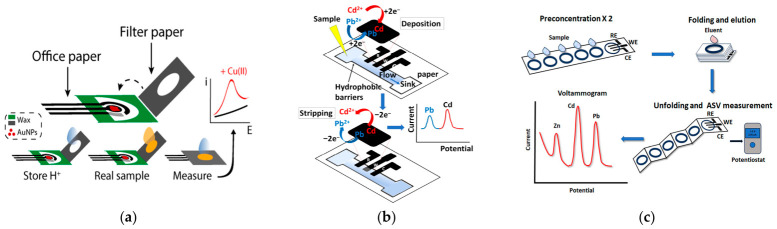
Examples of: (**a**) a first generation dual PAD for Cu(II) detection consisting of a disk made of filter paper for reagent storing and sample collection and 3-electrode ePAD patterned on office paper (reproduced from [87] with permission under CC-BY-NC-ND 4.0) (**b**) A second generation integrated PAD consisting of a fluidic channel with an overlaid screen-printed 3-electrode array for the determination of Pb(II) and Cd(II). (**c**) A third generation integrated folding PAD consisting of 5 preconcentration layers and a 3-electrode electrochemical cell for Cd(II), Pb(II) and Zn(II) detection (reproduced from [46] with permission under CC-BY-NC-ND 4.0).

The ePADs developed for stripping analysis of trace metals can be broadly categorized into three generations in terms of integration and functionality. The first generation involves a PAD used for sample collection which is physically attached to a separate home-made or commercial electrochemical sensing unit incorporating a set of three electrodes; the secondary sensing device can be patterned either on paper or a different type of substrate (e.g., on plastic). The second generation of ePADs are integrated devices that combine the fluidic sample conduit or assay zone and the electrochemical cell on the same paper-based substrate. Finally, the third generation of ePADs comprises integrated multi-layered and folding devices, sometimes with scope for bimodal detection, for increased functionality and ease of use. Examples of the three generations are illustrated in Figure 2.

Regarding the first generation, the first application was reported by Nie et al., who employed a paper fluidic device in contact with a plastic or paper substrate featuring screen-printed electrodes deposited in-house for the determination of Pb(II) [48]. Similar arrangements, based on the combination of commercial screen-printed three-electrode sensors with paper disks or fluidic devices, have been described by other workers for the determination of trace metals by stripping voltammetry [55,66,71,79] and As(III) by stripping amperometry [38]. The related work by Han et al. is noteworthy in that it uses a Sb_2_O_3_-modified working electrode [75]; the precursor is converted to metallic Sb during the analysis with enhanced sensitivity for Cd(II). A paper-based device with stored reagents was applied to adsorptive CSV (AdCSV) of Ni(II) [51]. Pungjunun et al. described an origami ePAD using an external AuNPs-modified working electrode for the speciation of arsenic [37]. Pang et al. have reported the fabrication of a carbon-paper electrode sequentially modified with AuNPs and a MOF; this was used as an external working transducer in a stacked portable arrangement for Pb(II) and Cd(II) detection [49]. Similar devices, featuring a carbon-paper electrode on ITO, were developed for ESA of heavy metals after modification with bismuth [63,64]. Bui et al. have described a carbon paper electrode modified with AuNPs-SeNPs for dual nitrate-Hg(II) assay [65]. Chailapakul’s group have fabricated a folding PAD with an externally attached BDD working electrode for Cu(II) analysis by adsorptive ASV (AdASV) [67]. An interesting paper-based device was developed by Ninwong et al. for quadruple metal detection [88]; the device exploits enhanced preconcentration efficiency induced by heating and was applied to Pb(II) and Cd(II) detection by ASV. Mettakoonpitak et al. have described a device with bimodal detection for oxidative potential and Cu(II) using a 1,10-phenanthroline/Nafion modified graphene screen-printed electrode using AdCSV as the detection technique [76]. Finally, Cinti’ s group have developed a Cu(II) sensor consisting of a chromatographic paper disk (to store reagents and collect samples) and a three-electrode cell screen-printed on office paper [87] (Figure 2a).

As far as the second generation of ePADs is concerned, a typical device is illustrated in Figure 2b. Ruecha et al. have fabricated a screen-printed sensor featuring a working electrode modified with a graphene–polyaniline nanocomposite for simultaneous detection of Zn(II), Cd(II), and Pb(II) [36]. Economou’s group have developed integrated ePADs using bismuth-modified screen-printed [44] or pen-plotted electrodes [45] for the determination of Pb(II) and Cd(II). A sustainable and inexpensive office paper-based electrochemical device was described for Zn(II) in serum [41]. The same group has reported on a paper-based sensor for Cu(II) determination in biological fluids [56]. Another interesting example involves sputtering of a three-electrode metal array on top of a fluidic device; the sputtered tin-film working electrode is suitable for Cd(II) and Zn(II) assays [68]. Iwuoha’s group have developed two three-electrode sensors by screen-printing Ag ink on photographic paper for Ni(II) determination by AdCSV: the first sensor featured a working electrode modified with carbon black-DMG [58], while the second one was based on rGO-AuNPs modification [80]. Arduini’s group have used a miniaturized paper-based screen-printed sensor, ex situ modified with bismuth, for Zn(II) detection after extraction with a 3D-printed chamber [57]. A screen-printed paper sensor modified with rGO-CeO_2_ was reported for As(III) in groundwater [81]. A paper-based sensor with synergistic action of rGO and in situ Bi plating was used for Cd(II) and Pb(II) determination [43] while a gold-sputtered three-electrode ePAD was applied to Cu(II) determination [42]. Chailapakul’s group have reported on an integrated ePAD for Pb(II) and Sn(II) monitoring using a graphene/BiNPs-modified working transducer [83]. The same group have developed a three-electrode sensor for Ni(II) [47] and a dual-mode method using an ESA sensor for Au(III) and colorimetric detection of Fe(III) [89]. Finally, a point-of need integrated sensor, that can be attached to sample vial lids, has been reported for heavy metal detection in water [60].

In the frame of the third generation of ePADs, an integrated multi-layer lateral-flow lab-on-a chip device with filtering ability has been developed for trace metals determination [39]. Silva-Neto et al. have described a plug-and-play folding device combining a paper-based ePAD and a fluidic PAD for multiplexed heavy metals determination [77]. A multi-folding paper-based device has been developed integrating five assay zones for metal preconcentration and an electrochemical cell; the targets were vertically eluted and detected with enhanced sensitivity at a bismuth citrate-modified working transducer [46] (Figure 2c). A similar arrangement was described for Hg(II) detection [74]. Henry’ s group have fabricated a Janus ePAD featuring four cells for simultaneous detection of Cd, Pb, and Cu by ASV and Fe and Ni by AdCSV in a single sample [50]. The same group have reported an electrochemical-optical microfluidic three-dimensional paper-based PAD for dual-mode colorimetric and ESA detection of toxic metals [40].

Organic compounds have also been determined by ESA on paper-based devices. An innovative application was reported for the in situ fabrication of a two-carbon electrodes array on paper by laser ablation [62]; the sensor was used for uric acid detection by ESA. In another piece of work, mitoxantrone was quantified by AdCSV at an ePAD featuring a screen-printed sensor modified with MB/Ti_3_C_2_T_x_/MWCNTs [82].

## 5. Biosensing at PADs with ESA

In the context of biosensing, ESA can be coupled with biological recognition mechanisms to achieve high selectivity [23,90,91]. Biorecognition elements such as DNA, aptamers, or antibodies can selectively bind target analytes and facilitate their accumulation on the electrode or paper surface [32]. In biosensor design, ESA is particularly attractive because it can be seamlessly integrated with these biorecognition elements whereby the specific interaction between the target analyte and the bioreceptor enhances selectivity. In the context of biosensing, this approach is often implemented indirectly: nanomaterials such as metal nanoparticles [59,91,92,93] or metal-based quantum dots (QDs) [69,94] serve as redox signal tags and the biological recognition event is translated into the release of the metal labels which are then quantified by ESA (Figure 1B,C). The use of multiple metal “barcoding” labels, especially QDs, with distinct stripping potentials enables multiplexed detection of several biotargets within a single paper-based platform [23,90,91]. Signal amplification strategies and enzymatic metal deposition are also often employed to further enhance sensitivity [95]. Overall, PAD biosensors employing ESA represent a highly sensitive and versatile analytical approach that bridges biological recognition with electrochemical amplification on a low-cost paper platform. Their ability to achieve laboratory-level sensitivity using disposable, field-deployable devices positions them as a promising technology for biosensing in resource-limited and decentralized settings.

Historically, the earliest applications of paper-based devices for biosensing using ESA involved lateral-flow strips (LFS’s) made of nitrocellulose. The first application was a duplex lateral flow assay for rabbit immunoglobulin G (IgG) and human immunoglobulin M (IgM) using AuNPs as labels [74]. The two test lines of the LFS were cut, transferred to a vial and the AuNPs were quantified by ASV at a commercial screen-printed sensor after acidic dilution [96]. Since this procedure is laborious and time consuming, in situ detection was performed by attaching a three-electrode sensor directly underneath the test line of a LFS; in this way, a novel sensitive immunochromatographic electrochemical biosensor for the detection of human chorionic gonadotropin (HCG) was developed [72]. Bi(III), coupled to the reporting antibody through a chelating agent (diethylenetriamine pentaacetic acid), was used as a signal tag; the acidically released Bi(III) was then detected by ASV on a mercury-coated working electrode. Similar arrangements exploiting CdS@ZnS QDs for prostate-specific antigen detection were developed, in which Cd(II) released from the QDs was ultimately detected [97,98].

A new ePAD was proposed for Botulinum neurotoxin A (BoNT/A) determination. SNAP-25 peptide, immobilized on a AuNPs/graphdiyne-modified paper-based electrode, was used to capture BoNT/A. Silver deposition on the modified electrode was catalytically induced and the Ag was measured by ESA [95].

Our group has developed an ePAD for the voltammetric determination of DNA [69]. The device was patterned by wax-printing on paper and featured an electrochemical cell with sputtered electrodes. The bioassay involved attachment of captured DNA, hybridization with biotinylated target oligonucleotide and labeling with streptavidin-conjugated CdSe/ZnS QDs. After the acidic dissolution of the QDs, the released Cd(II) was quantified by ASV at the sputtered tin working electrode. We have also described a bimodal QD- linked paper-based immunosensing platform for carcinoembryonic antigen (CEA) consisting of two separate PADs [99]. The first PAD was used for a sandwich immunoassay with CdS QDs labeling and in situ fluorescence detection. The second ePAD was used for ESA detection of Cd(II) released from the immunocomplex. An improved design of the same bimodal concept was recently developed for duplex detection of CEA and cancer antigen (CA125) [100]; in this case an integrated folding fluidic ePAD with two spatially separated assay zones was used.

Over the years, the Crooks’s group have developed a series of paper-based biosensors utilizing silver nanoparticles (AgNPs) as reporting probes; the AgNPs are oxidized and subsequently detected by ESA. Initially, they have described a generic paper-based biosensor (oSlip) in which the target analyte is labeled with AgNPs, which are magnetically preconcentrated at the detection electrode, oxidized by KMnO_4_ and detected by ASV [92]. Based on the same principle, a sandwich paper-based DNA metalloassay for the hepatitis B virus [93], and metalloimmunoassays for Trefoil Factor 3 [101] and NT-proBNP [59], were developed. Another metalloimmunoassay for ricin followed, this time using ClO^−^ as the oxidant [102]. An extension of this work was the *No*Slip design, based on galvanic exchange in which Au(III) reacted with the AgNPs to form Ag(I) and metallic Au; then, the Ag(I) was detected by ASV [103]. An improvement in the previous approach involved replacing AgNPs with Ag nanocubes with an associated increase in detection sensitivity [104].

## 6. Potentialities, Limitations and Future Prospects

Currently, no PADs using ESA are commercially available, an indication that they have some way to go before achieving the transition from proof-of-concept prototypes to user-friendly commercial products. The majority of sensors reported in the academic literature remain at low Technology Readiness Level (TRL), demonstrating proof-of-concept validation under controlled laboratory conditions, a maturity level way far from the higher TRL required for commercialization [105]. Bridging the gap between laboratory prototypes and industrial manufacturing platforms remains a significant challenge, both technically and economically.

General limitations of PADs that still impede their widespread adoption include [3]: the variability in paper pore size leading to difficulties in flow control; the non-specific adsorption of the analyte(s) and of potentially interfering species as well as matrix components that can induce matrix effects; the low sensitivity due to dissolution of dry reagents and the stronger retention of analytes in the paper matrix; the limited mechanical robustness of paper; the sample evaporation; limitations in using organic solvents and surfactants; the reproducibility in device fabrication; and the shelf-life, especially in devices with immobilized reagents and biomolecules. The implementation of electrochemical detection in PADs introduces additional challenges such as [5]: the incomplete incorporation of the conductive electrode material into the internal structures of the paper limiting the sensitivity; the variability in transducer fabrication; and difficulties related to miniaturization of electrochemical instrumentation [1].

Nevertheless, the synergy of ESA and PADs brings about some distinct advantages. Initially, this combination is highly versatile, being compatible with LFSs, multi-layered PADs, integrated PADs and folding paper-based configurations. Regarding trace metal analysis, this approach is ideal for determination of multiple target metals on the same platform, either at a single electrode or at spatially separated transducers. On the other hand, the high sensitivity of ESA allows extensive dilution of the sample, thus minimizing matrix effects. The combination of ESA with optical detection on a single platform can increase the reliability of measurements and the analytical dynamic range [99,100]. Finally, the use of multiple QDs as “barcode” tags enables multiplexed biosensing at a single working electrode by exploiting the distinct stripping potentials of the metals released from these QDs [100].

In addition, ongoing advances in materials science, microfabrication techniques, and device integration are steadily overcoming the aforementioned limitations [106]. The continued development of paper-based (bio)sensors employing ESA is expected to play a crucial role in enabling sensitive, affordable, and accessible analytical technologies for real-world applications, particularly in resource-limited and point-of-need settings. This transition is facilitated by developments in portable instrumentation [1,107,108] and paper-based electrochemical readers [109]. New functionalities are continuously introduced in PADs [106] that can extend their scope for sample manipulation and removal of matrix effects [1,110]. In addition, the gradual adoption of cellulosic materials derived from plant waste can lead to recycled paper sensors, thus contributing to the principles of sustainability and circular economy [111]. Finally, the integration of multiplexing strategies with microfluidic platforms and adaptive artificial intelligence (AI) algorithms can increase the real-time and high-throughput information content [112,113,114].

## Data Availability

No new data were created or analyzed in this study. Data sharing is not applicable to this article.

## Abbreviations

The following abbreviations are used in this manuscript:

AdASV	Adsorptive anodic stripping voltammetry
AdCSV	Adsorptive cathodic stripping voltammetry
AgNPs	Silver nanoparticles
AI	Artificial intelligence
ASV	Anodic stripping voltammetry
AuNPs	Gold nanoparticles
BDD	Boron-doped diamond
BoNT/A	Botulinum neurotoxin A
BiNPs	Bismuth nanoparticles
CB	Carbon black
CEA	Carcinoembryonic antigen
CNTs	Carbon nanotubes
CSV	Cathodic stripping voltammetry
DMG	Dimethylglyoxime
DNA	Deoxyribonucleic acid
ePAD	Electrochemical paper-based device
ESA	Electrochemical stripping analysis
GO	Graphene oxide
HCG	Human chorionic gonadotropin
IgM	Immunoglobulin M
IgG	ImmunoglobulinG
LFS	Lateral-flow strip
MOF	Metal-organic framework
MWCNTs	Multi-wall carbon nanotubes
PAD	Paper-based device
PANI	Polyaniline
PSA	Potentiometric stripping analysis
QDs	Quantum dots
rGO	Reduced graphene oxide
SeNPs	Selenium nanoparticles
